# Propagation and Maintenance of Cancer Stem Cells: A Major Influence of the Long Non-Coding RNA *H19*

**DOI:** 10.3390/cells9122613

**Published:** 2020-12-05

**Authors:** Clément Lecerf, Evodie Peperstraete, Xuefen Le Bourhis, Eric Adriaenssens

**Affiliations:** Univ. Lille, CNRS, INSERM, CHU Lille, UMR 9020 – UMR 1277 – Canther – Cancer Heterogeneity, Plasticity and Resistance to Therapies, F-59000 Lille, France; clement.lecerf@univ-lille.fr (C.L.); evodie.peperstraete@univ-lille.fr (E.P.); xuefen.le-bourhis@univ-lille.fr (X.L.B.)

**Keywords:** lncRNA, *H19*, non-coding RNA, cancer stem cells, tumorigenicity, reprogramming factors, exosomes

## Abstract

Cancer stem cells (CSCs) represent a rare population of tumor cells that exhibit stem cell properties with the abilities of self-renewal and differentiation. These cells are now widely accepted to be responsible for tumor initiation, development, resistance to conventional therapies, and recurrence. Thus, a better understanding of the molecular mechanisms involved in the control of CSCs is essential to improve patient management in terms of diagnostics and therapies. CSCs are regulated by signals of the tumor microenvironment as well as intrinsic genetic and epigenetic modulators. *H19*, the first identified lncRNA is involved in the development and progression of many different cancer types. Recently, *H19* has been demonstrated to be implicated in the regulation of CSCs in different types of cancers. The aim of this review is to provide an overview of the role and mechanisms of action of *H19* in the regulation of CSCs. We summarize how *H19* may regulate CSC division and cancer cell reprogramming, thus affecting metastasis and drug resistance. We also discuss the potential clinical implications of *H19*.

## 1. Introduction

Despite recent progress in early detection and therapeutics outcomes, cancer remains a major medical issue. Increasing data highlight the implication of cancer stem cells (CSCs) in tumorigenicity and cancer progression. CSC concept states that tumor growth, analogous to the renewal of healthy tissues, is fueled by a small number of dedicated stem cells according to a hierarchic model. At the top of the model, CSCs, through symmetric or asymmetric divisions, will give rise to self-renewal daughter CSCs and more differentiated transient amplifying cells to regenerate a heterogeneous tumor population. CSCs can be derived from adult normal stem/progenitor cells after (epi)genetic alterations. The mutated CSCs exhibit enhanced immune evasion capacity and reduced apoptosis, resulting in tumor initiation. Due to their unlimited division potential, CSCs will accumulate additional (epi)genetic alterations, leading to the appearance of diverse phenotypes of CSCs, tumor progression, and metastasis formation [1].

The evolving phenotype of CSCs is found to be tightly associated to the epithelial-to-mesenchymal transition (EMT) in cancers of epithelial origin [2,3,4]. EMT is a developmental process wherein epithelial cells transdifferentiate into mesenchymal cells. This process is characterized by molecular reprogramming, leading to cytoskeleton reorganization, cellular junction disruption, and increased abilities of cells to migrate and invade adjacent tissue. It was shown that tumor cells expressing high levels of the EMT master transcription factor SNAI1 display enhanced tumor-initiating capacity and metastatic potential in mouse and human models [4]. Similarly, another EMT inducer, ZEB1, was described to regulate the transit of basal breast cancer cells between non-CSC and CSC states [5]. In addition, the genetic reprogramming orchestrated by EMT affects intracellular mechanisms such as glucose, lipid, glutamine, and nucleotide metabolisms [6], sustaining the acquisition and maintenance of CSC characteristics.

Apart from EMT, differentiated cancer cells have been largely reported to be directly reprogrammed to CSCs by extracellular cues from the tumor microenvironment including hypoxia, ROS, and cytokines [7,8], as well as by ectopic expression of pluripotent transcriptional factors OCT3/4, SOX2, KLF4, and cMYC [9]. Moreover, radio- and chemotherapies are also able to increase CSCs by reprogramming mechanisms [10,11,12]. From a clinical point of view, this is of major importance, as CSCs present endogenous resistance mechanisms against radiation and chemotherapy, which confers CSCs as a survival advantage over differentiated counterparts. In addition, CSCs can generate various subclones, increasing the risk of a more resistant fraction after anti-cancer therapy [11].

Whatever the origins, CSCs are controlled by both extracellular stimuli common to normal stem cells (Wnt, hedgehog, Notch, and TGF-β) and specific to tumor microenvironment (cytokines, ROS, hypoxia). Intrinsic regulations including core stemness transcriptional factors, epigenetic alterations such as telomerase reactivation [13,14] and deregulated dynamics of chromatin (de)compaction lead to stemness-related gene expression and differentiation-related gene repression. To complexify the aforementioned interconnected extracellular and intracellular networks, it is increasingly described that non-coding RNAs including long non-coding RNAs and microRNAs contribute to the regulation of CSCs by various mechanisms [15,16,17].

In this review, we will first describe *H19* and its action in cancer development in a general way, and then overview how *H19* may regulate CSC division and cancer cell reprogramming. We will also discuss the potential clinical implications of *H19*.

## 2. The Long Non-Coding RNA *H19* and Its Pleiotropic Oncogenic Actions in Different Cancers

At the beginning of the 2000s, the Encyclopedia of DNA Elements (ENCODE) consortium showed that about 80% of the genome is transcribed into functional RNAs, but only 2% are translated into proteins [18,19,20]. From the results of this project, a “transcriptional background noise’’ therefore would exist, but also transcribed but not translated genes that produce non-coding RNAs (ncRNAs). These ncRNAs are classified according to their length in small ncRNAs (less than 200 nt) and long ncRNAs (more than 200 nt). Nowadays, about 20,000 lncRNAs have been identified and characterized [21]. They show classical features of mRNAs like transcription by RNA polymerase II, 5′ capping, 3′ polyadenylation, and splicing [22,23]. LncRNAs are described to be involved in both normal and pathological development, including cancer [24,25,26,27].

The long non-coding RNA *H19* is the first discovered lncRNA. The *H19* RNA is transcribed from the *H19* gene, which is subject to genomic imprinting and is maternally expressed [28]. The *H19* gene is located on the human 11p15.5 locus, near the *IGF2* (Insulin-like growth factor 2) gene. This gene is composed of 5 exons and codes for a mature RNA of 2.3 kb transcribed by the RNA polymerase II. The transcript is normally spliced, polyadenylated, capped, and exported to the cytosol. Brannan et al. showed that no protein is associated to this transcript, and proposed that *H19* could act as a non-coding RNA [29].

*H19* is highly expressed during embryogenesis and sharply downregulated after birth for the majority of adult tissues [30]. *H19* is found to play important roles during embryogenesis and normal tissue homeostasis. For instance, it has been described that loss of *H19* lncRNA in embryonic endothelial precursors and pre-hematopoietic stem cells (HSCs) results in failed HSC generation, indicating a pivotal role of *H19* in HSC formation from embryonic SCs [31]. Furthermore, *H19* is found to be preferentially expressed in long-term HSCs compared to short-term HSCs or multipotent progenitors in the adult blood system where *H19* is found to maintain HSCs quiescence [32]. Similarly, *H19* contributes to maintain the SC phenotype of normal prostate cells [33] and prevents adipogenesis of bone marrow SCs [34].

*H19* is widely described to be involved in tumorigenesis and cancer progression [35]. *H19* is overexpressed in both leukemia and different types of solid cancers including glioma, melanoma, lung adenocarcinoma, breast, ovarian, and prostate cancers, as well as cancers of digestive (tongue, stomach, colon, liver, pancreas) and urinary systems (kidney, bladder) [35]. *H19* favors tumorigenesis by promoting genomic stability [36], enabling replicative immortality [37] and sustaining cell growth [38,39], migration, and invasion, as well as increased resistance to chemotherapies [40,41,42]. Furthermore, *H19* is also reported to be able to promote angiogenesis and tumor inflammation as well as avoid immune suppression [43,44,45]. *H19* has been demonstrated to exert its oncogenic actions at transcriptional, post-transcriptional, and post-translational levels. At the transcriptional regulation level, *H19* can activate transcription factors like E2F1 in pancreatic ductal adenocarcinoma, leading to increased cell proliferation [46]; *H19* can also interact with the PRC2 complex to recruit it to its target genes promoters, leading to the modulation of chromatin condensation and the inaccessibility of gene promoters to transcription factors [47]. The post-transcriptional regulation by *H19* involves the contribution of microRNAs (miRNAs). Interestingly, the interaction of *H19* with miRNAs pathways is dual: on one hand, *H19* is able to act as a ‘‘sponge’’ to sequester miRNAs and impede their action [48]. On the other hand, *H19* is itself the precursor of the miR-675 [49], which will in turn regulate several targets, including the growth suppressors RB and RUNX1 [50,51,52]. Concerning the post-translational regulation, the action of *H19* has been spotted out outside the nucleus. Indeed, *H19* can physically interact with proteins like p53 to impair its activity in gastric cancer cells and promote cell proliferation [39].

During the past years, along with the fundamental and clinical data highlighting the implication of CSCs in tumorigenicity and cancer progression, more and more studies show that *H19* is able to regulate CSCs. In breast cancer, our team showed that both *H19* and its miR-675 are involved in CSC enrichment [53]. In the following sections, we will discuss how *H19* interconnects to the maze of CSCs.

## 3. The Long Non-Coding RNA *H19* Promotes Symmetric Renewal of CSCs

**Figure 1:**
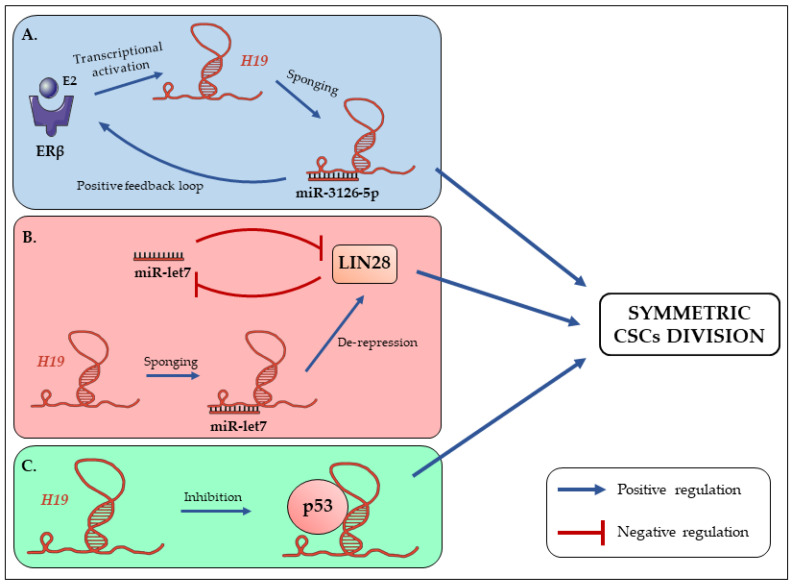
The long non-coding RNA *H19* promotes symmetric division of cancer stem cells (CSCs). (**A**) The activation of ERβ by the binding of its ligand (estradiol; E2) enhances *H19* expression, thus favoring miR-3126-5p sponging. *H19* can in turn promote ERβ to generate an activation loop [55,57]. (**B**) In breast CSCs, *H19* sponges let-7c and allows the de-repression of LIN28. The functional status and symmetric/asymmetric division of CSCs will thus be determined by the balance between *H19* and let-7 expressions [55,59]. (**C**) It has been shown that *H19* sequesters p53 to inhibit its activity and enhance gastric cancer cells proliferation [39]. Moreover, inactivation of p53 is associated with symmetric division of SCs [61]. We can thus hypothesize that *H19* could inactivate p53 to promote CSCs symmetric division.

As aforementioned, CSCs in a bulk tumor cell population may undergo asymmetric or symmetric divisions. Asymmetric division gives rise to a stem cell (SC) and a progenitor or committed cell (also called transient amplifying cell), while symmetric division leads to the generation of two identical daughter cells which are either CSCs (symmetric renewal) or committed cells [1]. Upon deregulated extrinsic and/or intrinsic cues, CSCs may preferentially undergo symmetric renewal to enlarge the pool of CSCs and sustain cancer progression [54]. In this way, *H19* is found to promote the symmetric renewal of CSCs through the regulation of several intrinsic intermediates including let-7, LIN28, or p53 [55]. Moreover, CSC symmetric renewal activity of *H19* is further amplified by the existence of positive regulatory loops between *H19* and extrinsic actors such as estrogen receptor β. For instance, estradiol (E2) treatment enhances *H19* expression in breast cancer cells [55,56]. *H19* will in turn promote the upregulation of estrogen receptor β (ERβ) expression in these cells [55]. Similar data have been shown in papillary thyroid carcinoma cells in which estradiol (E2) significantly promotes *H19* transcription via ERβ and elevates *H19* expression. On the other hand, *H19* acts as a competitive RNA to sequester miR-3126-5p, leading to enhanced ERβ expression. Depletion of *H19* reverses E2-induced stem-like properties, indicating the importance of the positive feedback loop in the enrichment of papillary thyroid carcinoma stem cells [57] (Figure 1A). In these models, the enhanced ERβ activity will thus promote symmetric division of CSCs and perpetuate the CSC pool within the tumor.

To go further, the mechanisms underlying this process showed an involvement of the let-7 miRNAs family. Let-7c inhibits the symmetric division and therefore stem-like phenotypes such as sphere-forming capacities of breast CSCs. Because of its overexpression due to enhanced Wnt signaling in this model, *H19* is able to sponge let-7c, thus allowing to counter its negative regulation and promote symmetric division of CSCs [55] (Figure 1B). Still in breast cancer, a similar mechanism has been highlighted: miR-146a expression indirectly upregulates let-7c to promote asymmetric division of breast CSCs. This goes through the post-translational targeting and degradation of LIN28. LIN28 is a transcription factor that can regulate gene expression either by binding to mRNAs or by blocking miRNA biogenesis, especially for the let-7 family [58]. Here, the degradation of LIN28 by miR-146a decreases Wnt signaling activation, and represses *H19* expression. Let-7c upregulation will thus block the symmetric division of breast CSCs that will in turn impact the CSCs pool [59] (Figure 1B).

One other thing concerning the symmetrical division of CSCs is the implication of p53 protein. The *TP53* gene codes for a protein involved in many cellular processes, hence its name of ‘‘cellular regulation platform’’. Indeed, p53 is found to act in response to several stresses such as DNA damage to regulate cell cycle, senescence, apoptosis, or genetic stability. Linked to this, it has been shown that SCs are resistant to DNA damage-induced apoptosis or senescence. This is notably due to the inactivation of p53 protein and activation of DNA repairing mechanisms [60]. The inactivation of p53 leads to cell cycle entry and symmetric division of SCs [61], and in the longer term might favor accumulation of SCs harboring DNA mutations, which can reduce their functional efficiency (aging) [62], and even favor transformation of normal SCs into CSCs.

In addition, it has been shown that *H19* is able to inhibit p53 activity, leading to the upregulation of gastric cancer cells proliferation [39]. Moreover, CSC treatment with a p53 pathway activator stimulates the asymmetric division [55]. It is thus possible that *H19* could promote symmetric divisions of CSCs by inhibiting p53 (Figure 1C).

## 4. *H19* Contributes to the Enrichment and Maintenance of CSCs

The origin of SCs has been studied as part of the cell reprogramming mechanism. Indeed, it has been shown that the transfection of transcription factors like *Oct4*, *Sox2*, *Klf4*, and *c-Myc* is sufficient to induce de-differentiation of murine fibroblasts. These fibroblasts are then reprogrammed to a pluripotent state that express a stem cell-like phenotype [63]. The phenotypic characterization of these induced-pluripotent stem (IPS) cells showed similar morphologic and growth properties when compared to embryonic SCs, and confirmed the expression of SC marker genes. In addition, authors showed that these IPS cells transplanted into nude mice are able to give rise to heterogeneous tumors. The reprogramming process starts with a set of cellular divisions, followed by epigenetic alterations: this is favored by little or no activity of caretakers genes such as *TP53* [64]. Among those epigenetic alterations, the influence of microenvironment through radiotherapy-induced reactive oxygen species (ROS) has been established [12,65]. Indeed, ROS activity leads to variations in the concentration of metabolic intermediates that are essential for histones post-translational modification, thus modulating the chromatin compaction level and so, gene expression.

It is well known in the literature that *H19* is functionally involved in the regulation of both SCs and CSCs. For instance, *H19* is found to maintain hematopoietic SCs quiescence, a mechanism in favor of the SC state. Indeed, the differentially methylated region (DMR) upstream of *H19* regulates the reciprocal expression of *H19* from the maternal allele and *Igf2* from the paternal allele. Deletion of the maternal but not the paternal H19-DMR alters hematopoietic SC quiescence and function [32]. Furthermore, *H19* expression is negatively correlated with adipocyte differentiation; conversely, overexpression of *H19* in bone marrow SCs prevents adipogenesis through post-translational inhibition of histone deacetylases (HDACs) 4-6 [34]. In prostate cancer, *H19* overexpression has been positively correlated with the expression of the well-known stemness-related factors *Oct4* and *Sox2*, and with cellular sphere-forming capacity [33] Furthermore, *H19* expression has been shown to be higher in papillary thyroid CSCs enriched by sphere formation than in monolayer cells. Moreover, these spheroid cells are characterized by both enhanced expression of *Nanog* and *Sox2* and reduced expression of differentiation markers. This suggests that *H19* is able to promote the reprogramming process, leading to the increase of cellular stemness [57].

The long non-coding RNA *H19* also participates to the maintenance of the CSC pool. For instance, microarray analysis showed an increased expression of *H19* and the pluripotent transcriptional factors Sox2, Oct4, and Nanog in the bulk acute lymphoblastic leukemia cells compared to early progenitors [66]. In addition, our team recently highlighted a stem cell gene signature (Aldh1a1+; CD44+/CD24−) in breast tumors expressing higher *H19*. Moreover, these gene signatures were also associated with enhanced *H19*-derived miR-675 expression, suggesting a role for both *H19* and miR-675 in the enrichment of breast CSCs [53].

**Figure 2:**
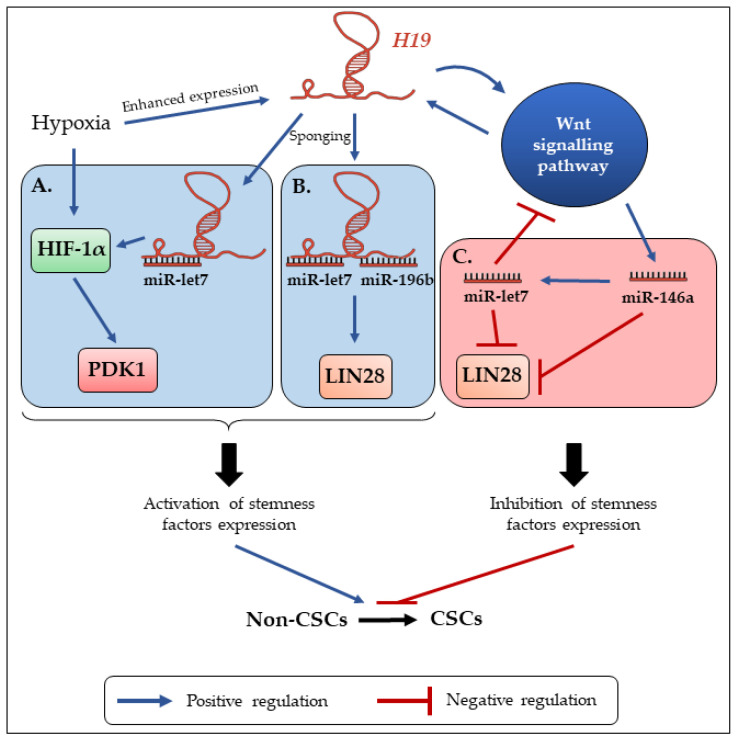
The balance between *H19* and let-7 expression controls the reprogramming of non-CSCs into CSCs. (**A**) Under hypoxic conditions HIF-1α expression is enhanced. Combined with the sponging of let-7 by *H19*, this leads to the enhanced expression of PDK1, which will in turn promote glycogenesis and expression of stemness phenotype [69]. (**B**) In breast and lung cancers, *H19* enhances the expression of the reprogramming factor LIN28 through the sponging of let-7 and miR-196b respectively [67,69]. (**C**) In breast cancer, the activation of the Wnt signaling pathway increases miR-146a production, which in turn promotes let-7 expression and so, the inhibition of reprogramming factors [55,59].

However, many studies describe the involvement of *H19* in reprogramming mainly through its role of miRNA sponge. Of interest, the above described loop regulation between *H19* and LIN28 in the promotion of CSCs symmetric division is also involved in reprogramming: indeed, in patient lung cancer samples, there exists a positive correlation between *H19* and LIN28 expressions, and further analysis showed an increase of LIN28 expression by *H19*. This is due to the sponging of miR-196b by *H19*, that normally supresses LIN28 mRNA translation. LIN28 expression allowed by *H19* leads to the promotion of lung cancer cell proliferation [67]. Similarly, *H19* has been found to sponge miR-3126-5p to allow the expression of ERβ receptor in papillary thyroid carcinoma, but also to sponge miR-193b in hepatocellular carcinoma, leading to the activation of MAPK1 and other oncogenes [57,68]. Furthermore, the complex regulation of miR-let-7 by *H19* can be also found involved in breast CSC reprogramming, echoing the regulation loop involved in CSC (a)symmetric division (Figure 2). Under hypoxic condition (which reflects intra-tumoral conditions), the expression of both HIF-1α and *H19* is enhanced. One of the downstream targets of HIF-1α is the *PDK1* gene, that codes for a glycolytic enzyme. PDK1 protein promotes glycolysis in breast CSCs, which is demonstrated to maintain cellular stemness with enhanced expression of reprogramming factors (*Oct4*, *Lin28*), enhanced ALDH1 activity and sphere-forming capacity (Figure 2A). However, in this model, HIF-1α expression was repressed. Further analysis showed that *HIF-1α* mRNA possesses in its 3′ UTR sequence a miR-let-7 response element: let-7 was thus responsible for HIF-1α degradation and low stem-like phenotypes in breast cancer cells. The authors showed that *H19* is able to sponge let-7 in order to overcome these effects, leading to the upregulation of HIF-1α and PDK1, contributing to breast CSC maintenance [69] (Figure 2A).

Still, in breast CSCs subpopulation, *H19* enhances ALDH1 activity, clone-forming, and sphere-forming capacities. These phenomena involve once again the enhancing of reprogramming factor LIN28 expression. To induce LIN28 expression, *H19* has to act as a competing endogenous RNA and sponge miR-let-7 (Figure 2B). However, it has been shown that LIN28 also suppresses let-7 production, and that *H19* can be downregulated by its own target let-7 in breast cancer cells. The authors therefore hypothesized that a cellular accumulation of LIN28 can indirectly enhance *H19* expression through inhibiting let-7 production. This highlights an interesting negative feedback loop involved in breast CSC maintenance. To reinforce this idea, this loop exhibits strong correlations in primary breast carcinomas [70]. In breast CSCs, the Wnt signaling pathway is strongly activated, leading to the enhanced expression of *H19*. Treatment of cells with Axitinib, a tyrosine kinase inhibitor used in clinical trials, revealed that the inhibition of Wnt activity was associated with both inhibition of *H19* expression and increase of asymmetric division rate [55]. However, in this model, Wnt signaling has been reported to be ‘‘naturally’ activated, as is *H19* expression. Further analysis has uncovered a positive feedback loop between *H19* expression and LIN28/let-7c axis. Indeed, Snail, a transcription factor involved in EMT, is able to indirectly promote miR-146a maturation through Wnt activity. In turn, miR-146a represses Wnt signaling activation through participating in the let-7c/Wnt/*H19* feedback loop (Figure 2C). Thus, the mechanisms involving miR-146a/Wnt and let-7c/Wnt contribute to form a complex and precise feedback loop of the miR-146a/let-7c/Wnt cascade. This feedback loop regulates the downstream expression of both let-7c and *H19*: the balance between these actors will thus determine the functional status of breast CSCs [59].

As described above (Figure 1B), increased let-7 production by miR-146a leads to the degradation of LIN28, and the downstream inhibition of the Wnt pathway, which in turn regulates *H19* expression [55,59] (Figure 2C). Thus, depending of the functional status of *H19*, this double regulation loop influences the CSC expansion. Sponging of let-7 by *H19* has also been described in glioblastoma. In this model, the inhibition of let-7 leads to the enhanced expression of its target HMGA2, which acts as an oncogene in several cancers. Re-expression of HMGA2 enhances mesenchymal transition of glioblastoma and self-renewal of glioblastoma SCs. For further investigations, the authors used phenformin, a mitochondrial complex I inhibitor used to inhibit cell growth and induce apoptosis of glioblastoma SCs. They showed that phenformin increases let-7 expression and thus represses *H19*-mediated stem-like phenotypes due to HMGA2 inhibition [71].

In other models such as hepatocellular carcinoma, *H19* has been demonstrated to induce both stemness and EMT to accelerate invasion of hepatocellular carcinoma cells in vitro. Indeed, as described above, it is known that EMT plays an important role in both inducing CSC characteristics and promoting cellular resistance to treatment [72,73]. In this model, *H19* sponges miR-193b, which will promote MAPK1 expression and de-repress several oncogenes like EGFR, KRAS, PTEN, and IGF1R. This is associated with enhanced expression of stemness genes such as *Lin28*, *Sox2*, *Notch1*, *Nanog*, and *Oct4.* All these events will thus trigger hepatocellular carcinoma progression and initiate the metastatic development [68]. The role of *H19* in the balance between both epithelial and mesenchymal phenotypes has also been shown by our team in the breast cancer model: thus, the mechanisms described in hepatocellular carcinoma could be transposed in the regulation of breast CSCs [74].

## 5. *H19* Enhances Drug Resistance of CSCs

Drug resistance is a major cause of low recurrence-free survival in various cancers. Cancer cells chemoresistance is multifactorial: it involves key factors such as cell behavior and growth kinetics, tumor heterogeneity, physical obstacles, and tumor microenvironment. Applying theses therapeutic pressures to cancer cells can also lead to tumor functional and adaptive reorganization, in order to persist despite the treatment. Particularly, within the tumor, CSCs are inherently more resistant to chemotherapy treatments, and have been proven to contribute to cancer relapse. Indeed, both chemotherapy and radiotherapy may promote CSC self-renewal through cytokine production and DNA repair mechanisms [75,76,77]. This is mainly due to the responsibility of CSCs in tumor heterogeneity. Thus, a high cellular proportion expressing CSC markers is correlated to poor prognosis and low response to treatments [78,79].

In addition, much evidence shows that *H19* is involved in drug resistance. Overexpression of *H19* in many cancers is associated with acquired chemoresistance and cancer cell survival, involving various mechanisms of action such as oncogene expression, epigenetic gene silencing, enhanced cell proliferation, apoptosis inhibition, and metastasis [80,81,82,83]. Furthermore, links between *H19*, CSCs, and drug resistance have also been established. In prostate CSCs, *H19* expression has been shown to promote both resistance to the androgen deprivation therapy (ADT) and the induction of highly metastatic form of prostate cancer [66]. In glioblastoma, knockdown of *H19* expression leads to decreased cellular proliferation and a higher apoptotic rate after induction by chemotherapy (temozolomide). This is accompanied by a downregulation of the CSC markers CD133, *Nanog*, *Oct4*, and *Sox2*, and thus the loss of glioblastoma cellular stemness. This is the proof that *H19* expression reinforces both stemness and chemoresistance of glioblastoma cells [84]. Moreover, *H19* is also associated with the stemness of colorectal cancer cells. High *H19* expression rates are found in patient samples at different tumor node metastasis (TNM) stages, and correlated with chemoresistance of colorectal cancer cells in vitro and in vivo after treatment with oxaliplatin. Indeed, *H19* expression enhances the populations of ALDH1^high^ cancer cells, the sphere-forming capacity of colorectal cancer cells, and the expression of pluripotency transcription factors *Nanog*, *Oct4*, and *Sox2*. In addition, resistance to oxaliplatin in *H19* overexpressing cells has been reported to be further enhanced [85]. In hepatocellular carcinoma, MDR1 (multidrug resistance 1) and GST-π (glutathione S-transferase-π) high protein expression levels were detected in CD133+ CSCs, in association with an overexpression of *H19*. In these cell lines, *H19* has been shown to affect the degree of oxidative stress by reducing the reactive oxygen species (ROS) production. In this model, inhibition of *H19* expression reduces CD133+ CSC chemoresistance through the enhancement of ROS production, the promotion of cell apoptosis and the blocking of the MAPK/ERK signaling pathway [86]. In liver CSCs, another non-coding RNA named *CUDR* (cancer up-regulated drug resistant) has been reported to confer cellular resistance to doxorubicin treatment [87]. Consequently, to its expression and activation after doxorubicin treatment, *CUDR* is able to increase both proliferation and malignant transformation of liver CSCs. This goes through the association of both cyclin D1 and PTEN in an inactive trimeric complex. PTEN knockdown leads to increase in the binding capacity of *CUDR* to cyclin D1, thus forming an active *CUDR*/cyclin D1 dimer that demethylates *H19* promoter and the downstream increase of telomerase activity. However, *H19* has been reported to regulate telomerase activity according to cell context, as *H19* would rather function as a molecular chaperone promoting either the association or the dissociation of telomerase subunits (TERT and TERC) [88]. In any cases, the expression of *H19* due to the demethylation of its promoter leads to the enhancement of liver CSC activity, and thus the tumor resistance to doxorubicine treatment. *H19* expression will also influence the telomerase activity and thus the long-term self-renewal capacity of liver CSCs [37].

## 6. *H19* Expression Is Propagated in the Tumor Micro-Environment to Promote Stemness

In addition to the maintenance of CSCs population, *H19* is found to promote and spread cellular stemness within a tissue or an organism. To do so, a transport means is needed to propagate *H19* within the extracellular environment. One of the possible options is the use of intercellular communication through extracellular vesicles, particularly the production of exosomes. Exosomes are a specific type of microvesicles produced in the endosomal compartment. They are characterized by their fusion with the cell surface to directly release their content (including proteins, lipids, and RNAs) in the extracellular medium [89]. Exosomes are present in many tissues and can also be found in blood. Moreover, they are also released in vitro by cultured cells into their growth medium [90].

Recently, exosomes released from cancer cells have been proposed to play a role in cancer progression. For example, exosomes are able to promote metastasis in initiating pre-metastatic niches in various cancers [91,92,93]. Furthermore, it has been shown that cancer cells and cancer associated fibroblasts (CAFs) can secrete exosomes [94,95]. According to their tumoral or stromal origin, those exosomes contribute to the crosstalk between cancer cells and the tumor microenvironment. In that manner, exosomes can be considered as critical intermediaries in tumor progression and metastasis.

**Figure 3:**
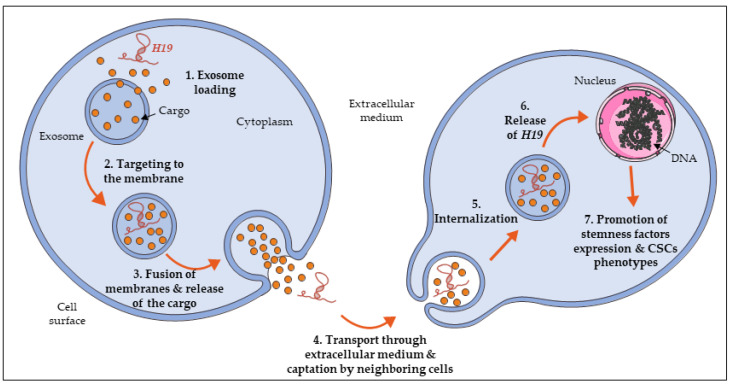
The long non-coding RNA *H19* disseminates into extracellular environment through exosomes, propagating its action in both normal and cancer cells.

Among the markers expressed by those exosomes, several molecules have been identified, including long non-coding RNAs like *H19* (Figure 3). Indeed, *H19* can act not only as an intrinsic factor, but can also act on neighboring cells. For instance, the involvement of the exosomes-derived expression of *H19* has been demonstrated in specific phenomena and pathologies such as trophoblast cell invasion, diabetic foot ulcers, and chronic cholestatic liver diseases, supporting our idea that exosomal *H19* can extend its action to surrounding cells [96,97,98]. In cancer models, it has been demonstrated that *H19* expression is upregulated in non-small cells lung cancer, particularly in gefitinib-resistant cells. Moreover, in these cells, *H19* is packaged into exosomes and secreted in the extracellular medium, to be then taken up by recipient cells and promote their gefitinib resistance [41].

Concerning the regulation of CSCs, it has been shown that colorectal cancer cells treated with conditioned medium derived from CAFs present an increase of sphere propagating capacity, cell viability, and activation of the Wnt/β-catenin pathway. More importantly, *H19* expression was found in CAFs-derived exosomes: this demonstrates the transport of *H19* from cells to others through exosomes [85]. In other models such as liver cancer, exosomes isolated from CD90+ cells promoted angiogenic phenotype and cell adhesion. CD90+ liver cancer cells are described as cancer stem cell-like, characterized with aggressive and metastatic phenotype. Further analysis of exosomes content showed an enrichment in *H19*, and that *H19* plays an major role in exosome-mediated phenotype of endothelial cells [99].

More importantly, it has been shown for bladder cancer diagnosis and prognosis that the concentration of circulating *H19* was significantly higher in serum exosomes than in exosomes-depleted supernatants in serum or tissue samples. Moreover, exosomes *H19* level was significantly increased in serum of bladder cancer patients when compared to healthy people or benign disease patients. Exosomes *H19* level was also correlated with poor survival. The detection of serum exosomal *H19* could thus be used as a new non-invasive diagnostic and prognostic biomarker for bladder cancer patients [100]. Combined with the proof of CSC regulation by *H19*, this highlights the importance of exosomal transportation of *H19* within the tumor.

## 7. Discussion

Both *H19* and *H19*-derived miR-675 are overexpressed in human bone marrow mesenchymal SCs. In this model, the downregulation of *H19* and miR-675 is correlated with the upregulation of IGFR1 (insulin-like growth factor receptor type 1) during neural differentiation [101]. miR-675 action in SCs is transposable in cancer as our team showed that miR-675 expression is associated with that of stem cell marker genes and that this miR is able to enhance stem-like phenotypes such as sphere-forming capacity [53]. From a clinical point of view, plasma levels of *H19* have been highlighted as predictive markers for breast, stomach, and lung cancers, but also as a way to follow the evolution of cancers [102,103,104]. Interestingly, the clinically approved medicine aspirin (acetylsalicylic acid) is able to inhibit *H19* expression and so repress expression of Oct4 and c-Myc in breast CSCs [69]. Similarly, *H19* is overexpressed in papillary thyroid carcinoma cells through estradiol (E2); *H19* overexpression is associated with increased stemness-related factors expression, increased ADLH+ population, and sphere forming capacity as well as enhanced tumor growth. In this model, aspirin attenuates E2-induced CSCs-like characteristics through decreasing both *H19* and ERβ expression.

**Figure 4:**
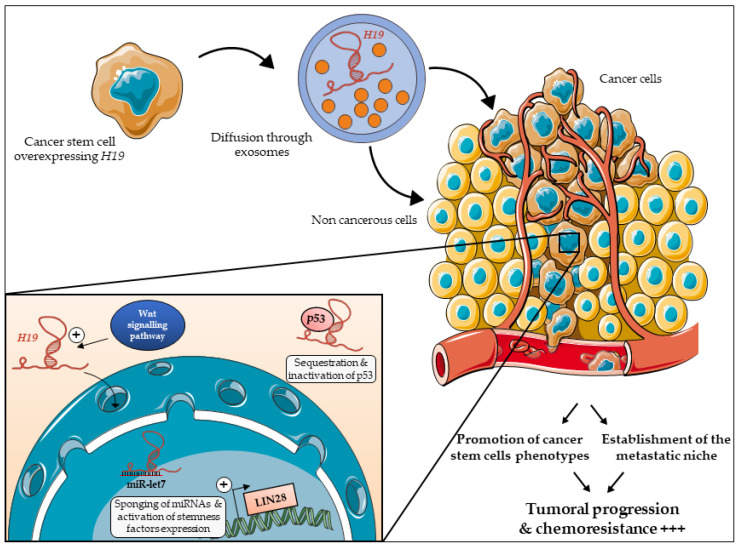
The long non-coding RNA *H19* promotes cancer stem cells phenotypes. *H19* is expressed by cancer stem cells and is exported to the extracellular medium through exosomes [85]. *H19* is then captured and internalized by surrounding cells. Within those cells, *H19* can act in different ways: *H19* can be translocated into the nucleus where it sponges miRNAs such as let-7. This allows the expression of reprogrammation factors and promotes stemness phenotypes [70]. In addition, *H19* can regulate the activity of factors such as p53 in order to promote symmetric division of CSCs [39,61]. Downstream, all these mechanisms will lead to the promotion of stem cells phenotype (symmetric division, reprogramming) in order to progress in the tumoral development and to reinforce CSCs’ resistance to therapies. Illustrations of this figure are from Servier Medical Art (https://smart.servier.com/).

The accumulated data demonstrating the role of *H19* and more recent findings of the involvement of miR-675 in CSC regulation complexify the regulatory network of CSCs (Figure 4). Further studies are warranted to verify if *H19* and miR-675 could be used as new markers of CSCs and therapeutic targets in the medical care of patients.

## Abbreviations

ADT	Androgen deprivation therapy
ALDH	Aldehyde dehydrogenase
BMP	Bone morphogenetic protein
CAF	Cancer-associated fibroblasts
CSC	Cancer stem cell
CUDR	Cancer up-regulated drug resistant
DMR	Differentially methylated region
EGFR	Epidermal growth factor receptor
EMT	Epidermal-to-mesenchymal transition
ERβ	Estrogen receptor β
GST-π	Glutathione S-transferase-π
HDAC	Histone deacetylase
HIF-1α	Hypoxia-inducible factor 1 alpha
HMGA2	High mobility group AT-hook 2
HSC	Hematopoietic stem cells
IGF1R	Insulin-like growth factor 1 receptor
IGF2	Insulin-like growth factor 2
IPS	Induced-pluripotent cells
KRAS	V-Ki-ras2 Kirsten rat sarcoma viral oncogene homolog
LncRNA	Long non-coding RNA
MDR1	Multidrug resistance 1
miRNA	MicroRNA
PDK1	Pyruvate dehydrogenase kinase 1
PRC2	Polycomb repressive complex 2
PTEN	Phosphatase and tensin homolog
RB	Retinoblastoma protein
ROS	Reactive oxygen species
RUNX1	Runt-related transcription factor 1
SC	Stem cell
TNM	Tumor node metastasis
